# Enantioselective Recognition of Chiral Carboxylic Acids by a β-Amino Acid and 1,10-Phenanthroline Based Chiral Fluorescent Sensor

**DOI:** 10.3390/s150510723

**Published:** 2015-05-06

**Authors:** Yonghong Zhang, Fangzhi Hu, Bin Wang, Xiaomei Zhang, Chenjiang Liu

**Affiliations:** 1 Key Laboratory of Petroleum and Gas Fine Chemicals of Ministry of Education, School of Chemistry and Chemical Engineering, Xinjiang University, Urumqi 830046, China; E-Mail: zhzhzyh@126.com; 2 Chengdu Institute of Organic Chemistry, Chinese Academy of Sciences, Chengdu 610041, China; E-Mails: ssfangzhi@126.com (F.H.); xmzhang@cioc.ac.cn (X.Z.); 3 Physics and Chemistry Detecting Center, Xinjiang University, Urumqi 830046, China; E-Mail: wangbin.yang@163.com

**Keywords:** enantioselective recognition, fluorescent sensor, carboxylic acids, 10-phenanthroline, β-amino acid

## Abstract

A novel chiral 1,10-phenanthroline-based fluorescent sensor was designed and synthesized from optical active β-amino acids. It used 1,10-phenanthroline moiety as a fluorescent signaling site and binding site, with optically active β-amino acids as a chiral barrier site. Notably, the optically active β-amino acids were obtained by a Lewis base catalyzed hydrosilylation of β-enamino esters according to our former work. The chiral sensor has been used to conduct the enantioselective recognition of chiral mono and dicarboxylic acids derivatives. Using this fluorescent sensor, a moderate “turn-off” fluorescence-diminishment response towards enantiomer of tartaric acids, and proline was observed. It found that l-enantiomers quench the chiral fluorescence sensor more efficiently than d-enantiomers due to the absolute configuration of the β-amino acid.

## 1. Introduction

Enantioselective recognition of the two enantiomers of chiral compounds is an important subject not only in the field of asymmetric catalysis systems but also in the field of medicinal and biomedical applications [1,2,3,4,5,6]. Among these subjects, enantioselective recognition of carboxylates has important implications in asymmetric synthesis and drug discovery in recent years [7,8,9,10,11,12,13,14,15,16,17,18,19]. Although traditionally analytical techniques such as NMR or gas/liquid chromatography have been commonly used to study the chirality of organic compounds in the chiral recognition [20,21,22], those methods have many disadvantages which impede their application such as time-consuming procedures, solvent waste, and being technically and instrumentally demanding. Accordingly, more and more attention has been paid to fluorescence-based chiral sensors due to their time-efficiency, accuracy, and sensitive enantiomeric determination of chiral reagents, catalysts, natural products and drugs [8,23,24,25,26]. However, to design and synthesize excellent fluorescent chiral receptors is still an urgent need in asymmetric synthesis and enantioselective recognition.

1,10-Phenanthroline has an extended planar aromatic structure and the ability to chelate metal ions that has played an important role in the development of coordination chemistry [27,28]. As a classic chelating bidentate ligand for transition metal ions, 1,10-phenanthroline derivates are well-known for their ability to form many useful metal complexes [29,30], and their chiral derivatives have been used as catalysis in enantioselective reactions that include ketone reduction [31], enolate allylation [32,33,34], oxidation [35], and cyclopropanation [36], among others [37,38]. However, chiral fluorescent sensors utilizing 1,10-phenanthroline moiety as a fluorescent signaling site or binding site have, to the best of our knowledge, not been reported, even though they have been widely utilized in catalysis in enantioselective reactions.

Optically active β-amino acids are very important chiral building blocks for the synthesis of β-peptides, β-lactams, natural products, and physiologically active substances [39]. In our former work [40,41], we synthesized β-amino acid derivatives in good enantioselective by the Lewis base catalyzed hydrosilylation of β-enamino esters. To further illustrate the synthetic potential of this methodology, herein, we describe the first general enantioselective recognition of carboxylic acids by a 1,10-phenanthroline-based fluorescent sensor, which synthesized from optical active β-amino acids derivatives.

## 2. Experimental Section

### 2.1. General

Chemicals were purchased from commercial suppliers and used without further purification unless otherwise stated. Solvents were dried and purified according to the standard procedures prior to use. Reactions were monitored by thin layer chromatography (TLC). Flash column chromatography was performed on silica gels (200–300 mesh).

### 2.2. Instrumentation

^1^H NMR and ^13^C NMR (300 and 75 MHz, respectively) spectra were recorded on a Bruker 300 MHz NMR spectrometer (Bruker, Fällanden, Switzerland) in CDCl_3_. ^1^H NMR chemical shifts are reported in ppm (δ) relative to tetramethylsilane (TMS) with the solvent resonance employed as the internal standard (CDCl_3_, δ 7.26 ppm). Data are reported as follows: chemical shift, multiplicity (s = singlet, brs = broad singlet, d = doublet, t = triplet, q = quartet, m = multiplet), coupling constants (Hz) and integration. ^13^C NMR chemical shifts are reported in ppm from tetramethylsilane (TMS) with the solvent resonance as the internal standard (CDCl_3_, δ 77.0 ppm). High Resolution Mass Spectromete (HRMS) data were obtained on BioTOF Q (Bruker, Karlsruhe, Germany). Optical rotations were measured on a Perkin-Elmer 241 Polarimeter (Perkin Elmer, Waltham, MA, USA). All enantiomeric ratios have been controlled by co-injections of the pure sample with the racemates. Fluorescence spectra were measured on a Hitachi F-7000 spectrophotometer (Hitachi, Tokyo, Japan) equipped with a 1 cm quartz cell. UV-visible spectra were acquired on a Techcomp UV1100 spectrophotometer (Techcomp, Shanghai, China).

### 2.3. Sample Preparations

#### 2.3.1. Synthesis of β-Amino Acid (***S*-10**)

**Scheme 1:**
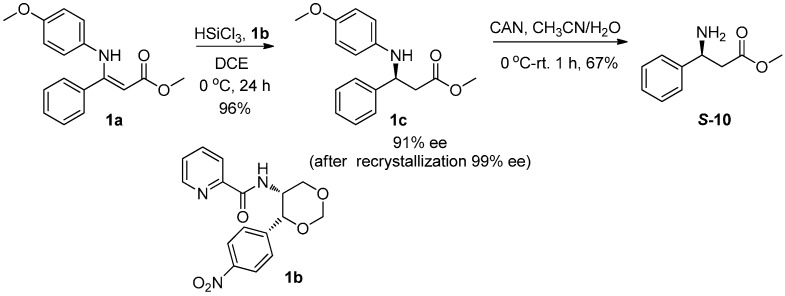
Synthesis of β-amino acids.

We efficiently synthesized β-amino acid ester **1c** via hydrosilylation of β-enamino ester **1a** by employing Lewis base **1b** as the catalyst. According to our former work [40], the reaction proceeded smoothly to provide **1c** in 96% yield and 91% ee. The ee values were enhanced up to optically pure after recrystallized with *n*-hexane, the *N*-PMP (PMP = *p*-methoxyphenyl) group of product **1c** was deprotected with CAN (CAN = ceric ammonium nitrate) to give β-amino ester ***S*-10** in 67% yield without racemization (Scheme 1).

#### 2.3.2. Synthesis of Fluorescent Chiral Sensor (***S*-G1**)

**Scheme 2:**
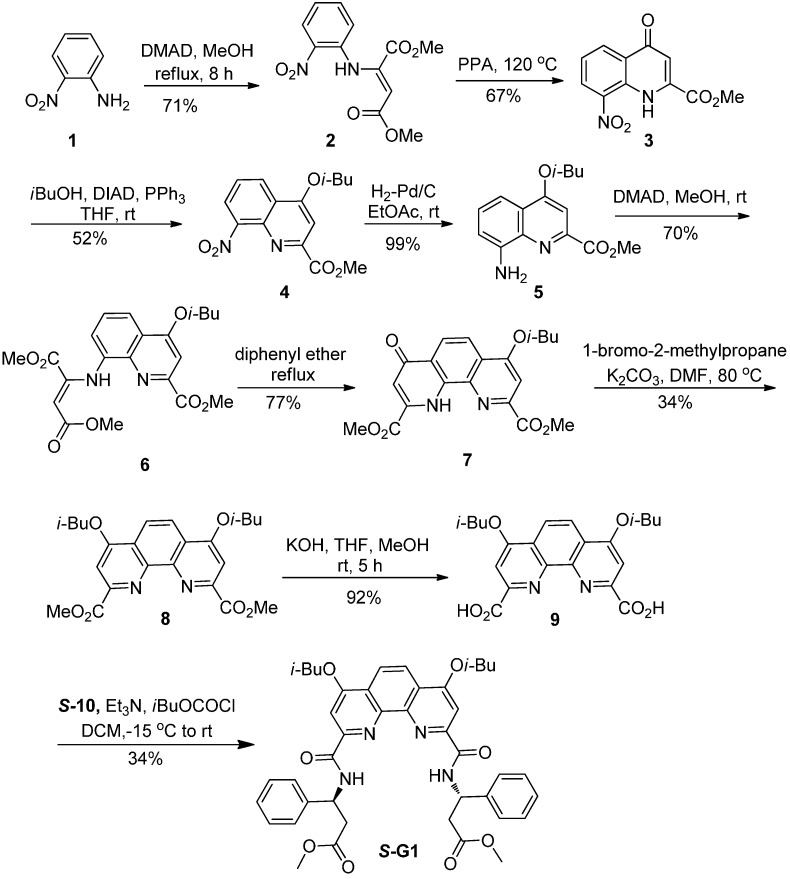
Synthesis of ***S*-G1**.

Chiral sensor (***S*-G1**) was prepared by coupled optical active β-amino acid ***S*-10** with 10-Phenanthroline derivative **9** (Scheme 2). Compound **9** was afforded through multi-step transformation with 2-nitroaniline and dimethyl acetylenedicarboxylate as starting materials according to the literature procedure [42,43].

##### Synthesis of ***S*-G1**

A 50 mL round bottom flask is charged with **9** (0.26 g, 0.5 mmol) and the flask is flushed with argon. It is dissolved in 5 mL of dry dichloromethane (DCM), and 0.43 mL of Et_3_N (3 mmol) is added. The flask is cooled to −15 °C with a NaCl/ice mixture and 0.2 mL of isobutylchloroformate (1.5 mmol) is added drop wise over 5 min. The mixture is stirred at this temperature for 40 min, 0.22 g of the β-amino acid derivative ***S*-10** (1 mmol) dissolved in 5 mL of dry DCM and 0.2 mL dry Et_3_N is added via a syringe. The bath is allowed to warm up to room temperature, and the reaction mixture is stirred at room temperature for 5 h. The contents are transferred to a separatory funnel and diluted with 10 mL of DCM. The organic layer is sequentially extracted with 1 N HCl (10 mL), water (10 mL), and brine (10 mL). It is then dried over Na_2_SO_4_ and the solvent was carefully removed under reduced pressure. The residue was directly subjected to column chromatography (petroleum ether/ethyl acetate = 5/1 as eluent) to afford ***S*-G1** 0.13 g (Yield 34%). Yellow solid; ^1^H NMR (CDCl_3_, 300 MHz, TMS): δ = 9.62 (d, *J* = 8.4 Hz, 2 H), 8.29 (s, 2 H), 7.97 (s, 2 H), 7.50–7.52 (m, 4 H), 7.21–7.34 (m, 6 H), 5.69–5.76 (m, 2 H), 4.10–4.16 (m, 4 H), 3.56 (s, 6 H), 3.00–3.18 (m, 4 H), 2.26–2.62 (m, 4 H), 1.14 (d, *J* = 6.7 Hz, 12 H); ^13^C NMR (CDCl_3_, 75 MHz): δ = 171.6, 164.3, 163.1, 151.1, 145.4, 141.0, 128.6, 127.5, 126.6, 122.7, 120.3, 102.1, 75.4, 51.9, 50.5, 40.1, 28.1, 19.2; HRMS (ESI) calculated for C_42_H_46_N_4_NaO_8_ 757.3248, found 757.3232.

### 2.4. Enantioselective Recognition of Chiral Carboxylic Acids with Fluorescent Chiral Sensor **S-G1**

The stock solutions of ***S***-**G1** and carboxylic acids were prepared in ethanol. Fluorescent measurements were carried out by exciting at 330 nm with an excitation and emission band width of 2.5 nm in the 8 × 10^−5^ mol·L^−1^ ethanol solution.

## 3. Results and Discussion

We design ***S***-**G1** utilizing 1,10-phenanthroline moiety as a fluorescence signaling unit and optically active β-amino acids as a chiral barrier site to undertake the desired push-pull type fluorescent sensor to recognition of carboxylic acids for the following reasons: (1) 1,10-Phenanthroline was used as a fluorophore core and the scaffold of the chiral sensors; (2) The nitrogen atoms of 1,10-phenanthroline as a binding site that should bind -COOH of chiral carboxylic acids well through multiple hydrogen bonds; (3) The optical active β-amino acids as the chirogenic barrier site that may lead to good chiral recognition; (4) When sensors interact with the chiral carboxylic acids, the fluorescence of 1,10-phenanthroline expected to be turned off through nonradiative relaxation of the sensor and the carboxylic acids complexes.

**Figure 1:**
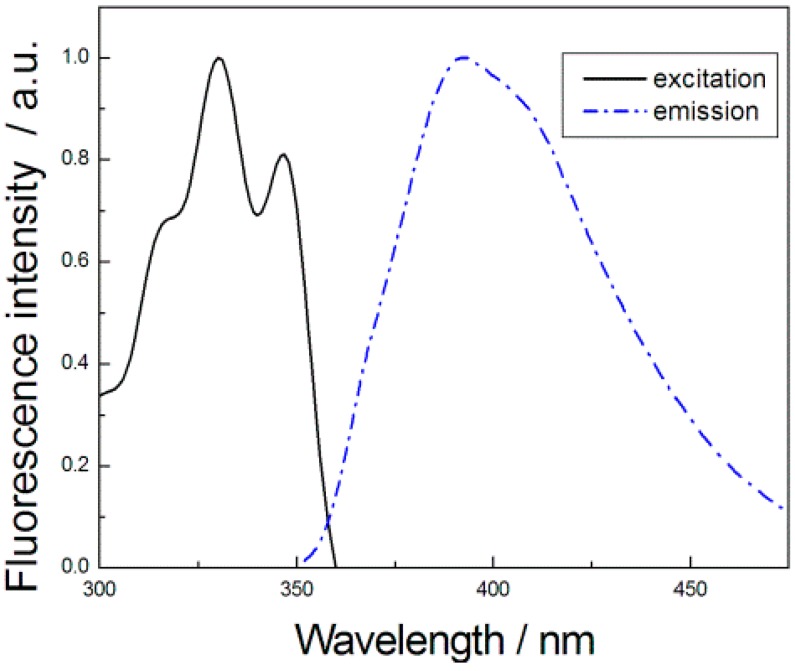
Normalized excitation and emission spectra of ***S*-G1** (λ_ex_ = 330 nm, λ_em_ = 392 nm).

The UV-vis and the emission spectra of ***S*-G1** were investigated (Figure 1). The solid line represents the UV spectrum of ***S*-G1** (8 × 10^−5^ M) in ethanol, and the dashed line represents the fluorescence spectrum of ***S*-G1** (8 × 10^−5^ M) in ethanol. The excitation and emission maxima are located at 330 (more accessible visible) and 392 nm, respectively. The Stokes shift is 62 nm.

Firstly, we investigated the fluorescence responses of ***S*-G1** in the absence and presence of both enantiomers of tartaric acids. An ethanol solution of ***S*-G1** (8 × 10^−5^ mol·L^−1^) was treated with the individual enantiomers of tartaric acid over the concentration range 8 × 10^−5^–1.2 × 10^−3^ mol·L^−1^.

**Figure 2:**
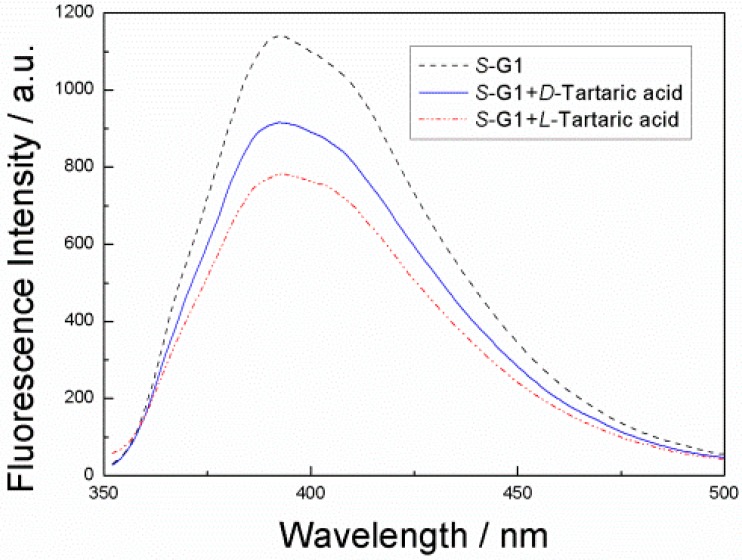
Fluorescence spectra of ***S*-G1** (8 × 10^−5^ mol·L^−1^) in EtOH solution, ***S*-G1** (8 × 10^−5^ mol·L^−1^) with d-tartaric acid (1.2 × 10^−2^ mol·L^−1^) and ***S*-G1** (8 × 10^−5^ mol·L^−1^) with l-tartaric acid (1.2 × 10^−2^ mol·L^−1^) (λ_ex_ = 330 nm) in EtOH solution.

As shown in Figure 2, both of the enantiomer tartaric acid quenched the fluorescence intensity. However, when ***S*-G1** is treated with l-enantiomers, a large fluorescence quenching is observed. When ***S*-G1** is treated with d-enantiomers, a slight fluorescence quenching is observed. In the concentration range studied, the fluorescence quenching follows the Stern–Völmer equation:

I_0_/I = 1 + K_SV_[Q]

where I_0_ is the fluorescence intensity in the absence of a quencher and I is the fluorescence intensity in the presence of a quencher. [Q] is the quencher concentration. K_SV_ is the Stern–Völmer constant, which measures the efficiency of quenching.

**Figure 3:**
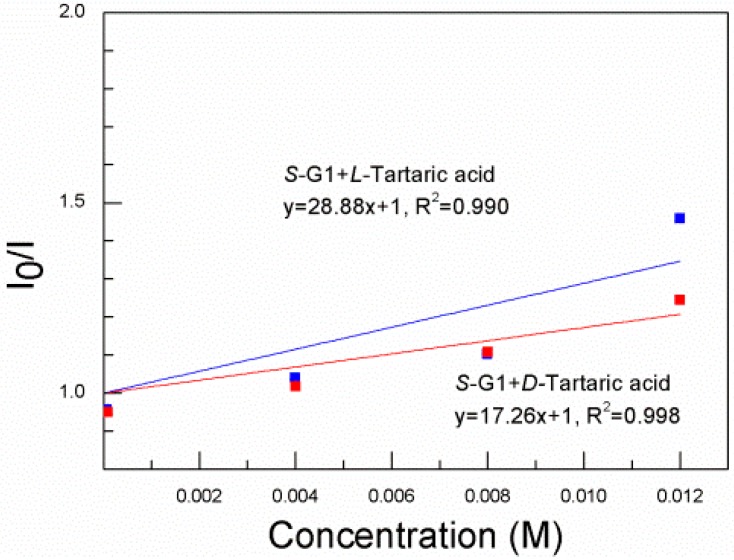
Stern–Völmer plot of ***S*-G1** (8 × 10^−5^ mol·L^−1^) in EtOH in the presence of d-tartaric acid and l-tartaric acid (λ_ex_ = 330 nm).

Figure 3 shows the Stern–Völmer plot of ***S*-G1** (8 × 10^−5^ mol·L^−1^) in the presence of d- and l-tartaric acid (8 × 10^−5^–1.2 × 10^−2^ mol·L^−1^) in EtOH. The Stern–Völmer constant of d-tartaric acid is 17.26 M^−1^(K_SV_^S,D^) in the presence of d-tartaric acid and 28.88 M^−1^(K_SV_^S,L^) in the presence of l-tartaric acid. The enantioselectivity is K_SV_^S,D^/K_SV_^S,L^ = 0.60. Thus, l-tartaric acid quenches the fluorescence of ***S*-G1** more efficiently than d-tartaric acid.

**Figure 4:**
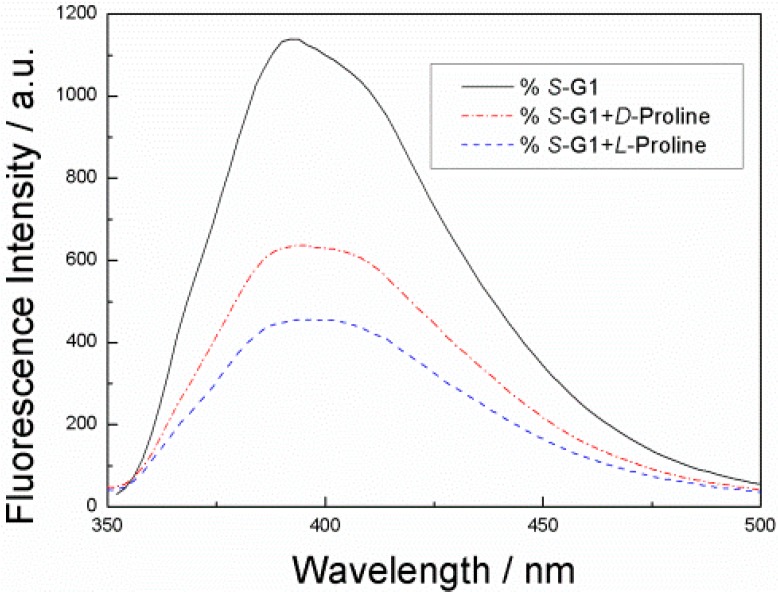
Fluorescence spectra of ***S*-G1** (8 × 10^−5^ mol·L^−1^) in EtOH solution, ***S*-G1** (8 × 10^−5^ mol·L^−1^) with d-priline (1.2 × 10^−2^ mol·L^−1^) and ***S*-G1** (8 × 10^−5^ mol·L^−1^) with l-proline (1.2 × 10^−2^ mol·L^−1^) (λ_ex_ = 330 nm) in EtOH solution.

The fluorescence responses of ***S*-G1** toward enantiomers of proline was also investigated in ethanol solution (8 × 10^−5^ mol·L^−1^) (Figure 4), which showed the similar enantioselectivity to tartaric acid. This confirms that the observed differences in the fluorescence quenching are due to chiral discrimination.

**Figure 5:**
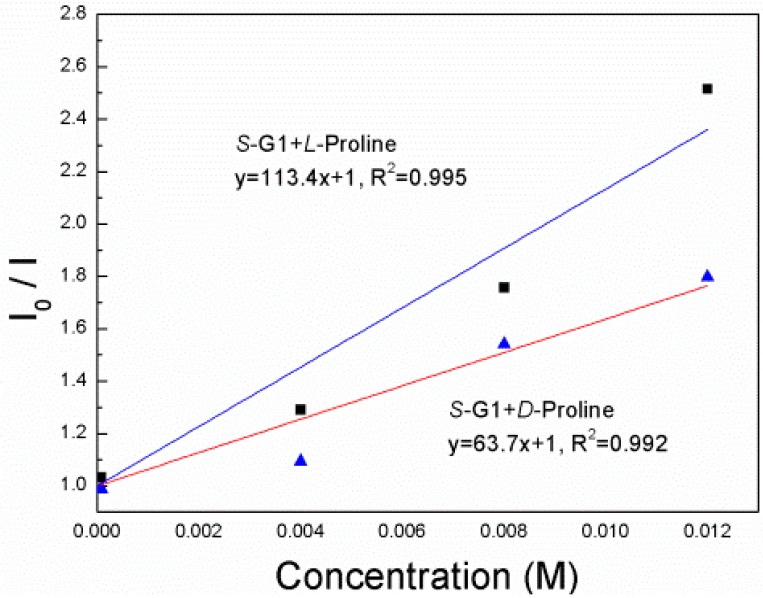
Stern–Völmer plot of ***S*-G1** (8 × 10^−5^ mol·L^−1^) in EtOH in the presence of d-proline and l-proline (λ_ex_ = 330 nm).

The Stern–Völmer constants of ***S*-G1** in the presence d- and l-Proline (Figure 5) were found to be 63.7 and 113.4 M^−1^, respectively, under the same conditions as the use of d- and l-Tartaric acid. The ratio K_SV_^S,D^/K_SV_^S,L^ is 0.56. Similar to enantiomers of tartaric acids, the fluorescence response of ***S*-G1** toward l-Proline acid is more significant than that with d-Proline.

The carboxylic acids induced fluorescence quenching of ***S*-G1** was probably attributed to a result of static quenching through nonradiative relaxation of diastereomeric acid-base adducts through hydrogen bonding. When sensors interact with the carboxylic acids through multiple hydrogen bonds, the fluorescence of 1,10-phenanthroline turn off through nonradiative relaxation of the sensor and the carboxylic acids complexes. At same time, the steric hindrance of the optical active β-amino acids leads to good chiral recognition.

## 4. Conclusions

In summary, we designed and synthesized a C2-symmetric 1,10-phenanthroline-bearing chiral fluorescent sensor ***S*-G1**. Enantioselective recognition of tartaric acids and proline were observed with this fluorescent sensor. l-enantiomers quench the fluorescence of ***S*-G1** more efficiently than d-enantiomers due to the absolute configuration of the β-amino acid. Consecutive fluorescence emission diminishment was observed with increasing the concentration of the tartaric acids or proline. Notably, a more accessible visible wavelength (330 nm) was used as excitation wavelength in this fluorescent sensor, which indicated its promising ability in biomedical applications.

## Supplementary Materials

Supplementary materials can be accessed at: http://www.mdpi.com/1424-8220/15/5/10723/s1.
