# Supplementary material for: Enantioselective Recognition of Chiral Carboxylic Acids by a β-Amino Acid and 1,10-Phenanthroline Based Chiral Fluorescent Sensor
Source: Sensors (Basel). 2015 May 6;15(5):10723–33. doi: 10.3390/s150510723 (PMC4481974; doi:10.3390/s150510723)
Supplement: Supplementary file 1 [file sensors-15-10723-s001.pdf]

OPEN ACCESS

sensors

ISSN 1424-8220

www.mdpi.com/journal/sensors

Supplementary Information

Enantioselective Recognition of Chiral Carboxylic Acids by a β-Amino Acid and 1,10-Phenanthroline Based Chiral Fluorescent Sensor. *Sensors* 2015, *15*, 10723-10733

Yonghong Zhang [1], Fangzhi Hu [2], Bin Wang [3], Xiaomei Zhang [2] and Chenjiang Liu [1,3,*]

[1] Key Laboratory of Petroleum and Gas Fine Chemicals of Ministry of Education, School of Chemistry and Chemical Engineering, Xinjiang University, Urumqi 830046, China; E-Mail: zhzhzyh@126.com

[2] Chengdu Institute of Organic Chemistry, Chinese Academy of Sciences, Chengdu 610041, China; E-Mails: ssfangzhi@126.com (F.H.); xmzhang@cioc.ac.cn (X.Z.)

[3] Physics and Chemistry Detecting Center, Xinjiang University, Urumqi 830046, China; E-Mail: wangbin.yang@163.com

* Author to whom correspondence should be addressed; E-Mail: pxylcj@126.com; Tel. +86-991-8581-211; Fax: +86-991-8582-809.

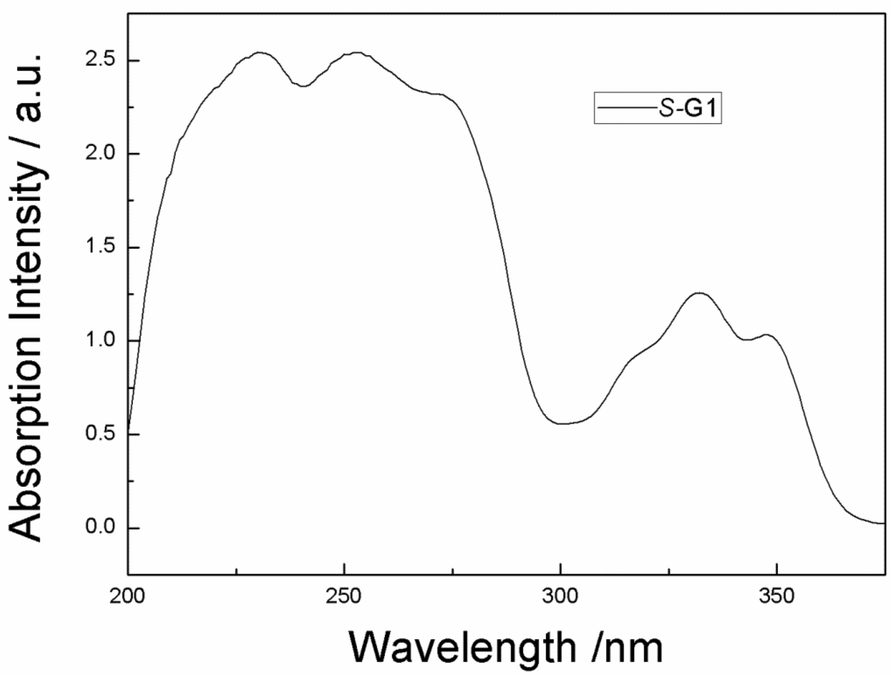

Figure S1. UV-Vis spectra of ***S*-G1** (8×10^{-5} mol/L) in a solution of EtOH.

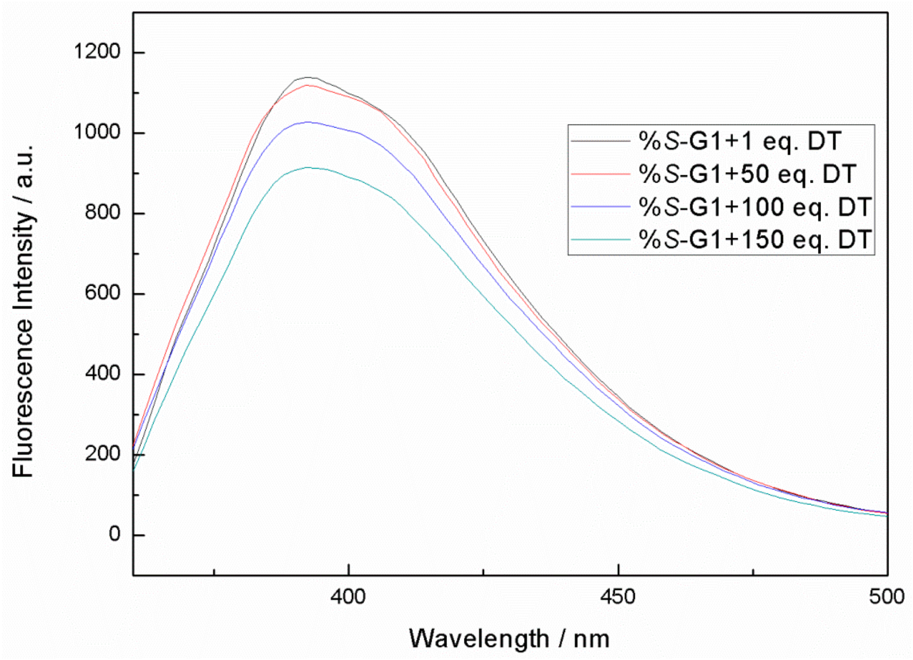

Figure S2. Fluorescence of ***S*-G1** (8×10^{-5} mol/L) in EtOH *versus* the concentration of D-tartaric acids (λ_{ex} = 330 nm).

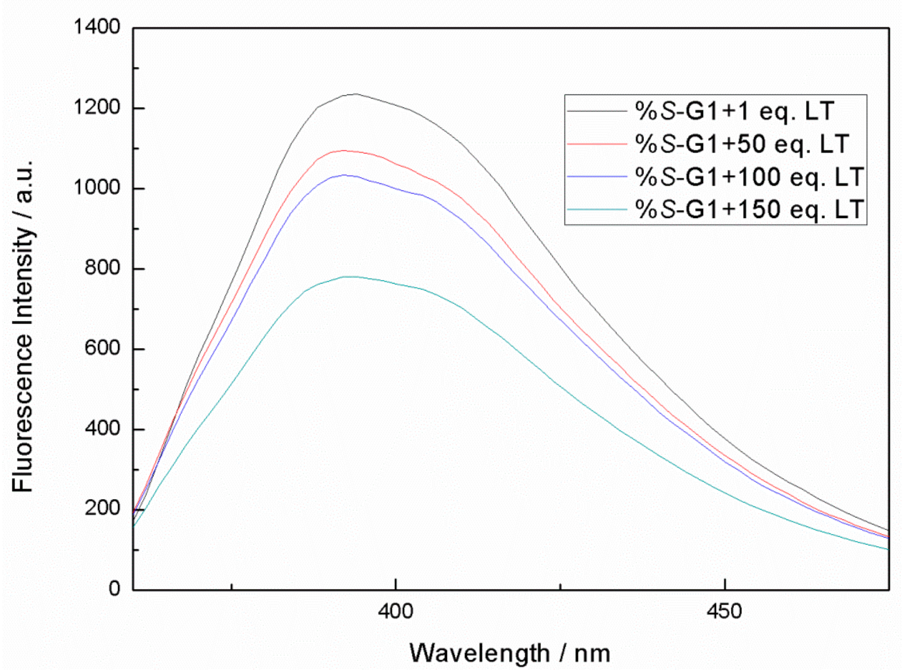

Figure S3. Fluorescence of ***S*-G1** (8×10^{-5} mol/L) in EtOH *versus* the concentration of L-tartaric acids (λ_{ex} = 330 nm).

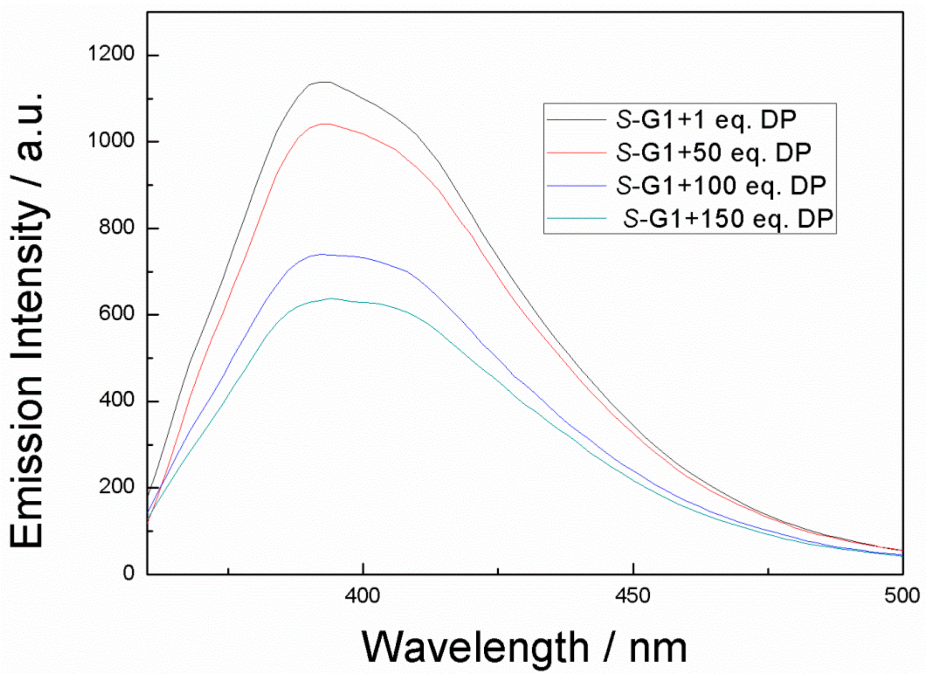

Figure S4. Fluorescence of ***S*-G1** (8×10^{-5} mol/L) in EtOH *versus* the concentration of D-proline (λ_{ex}= 330 nm).

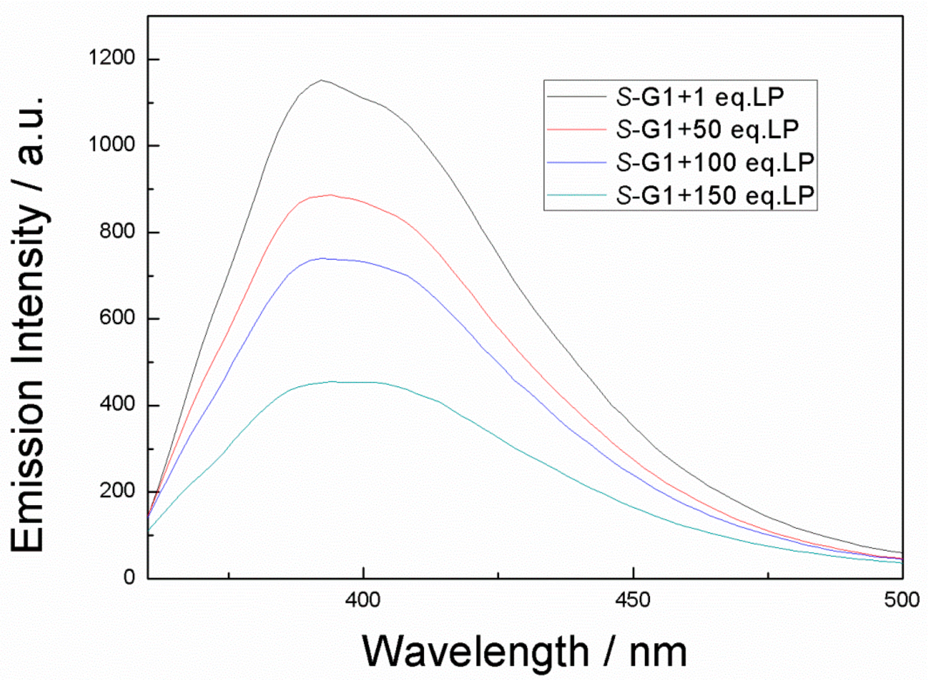

Figure S5. Fluorescence of ***S*-G1** (8×10^{-5} mol/L) in EtOH *versus* the concentration of L-proline (λ_{ex}= 330 nm).

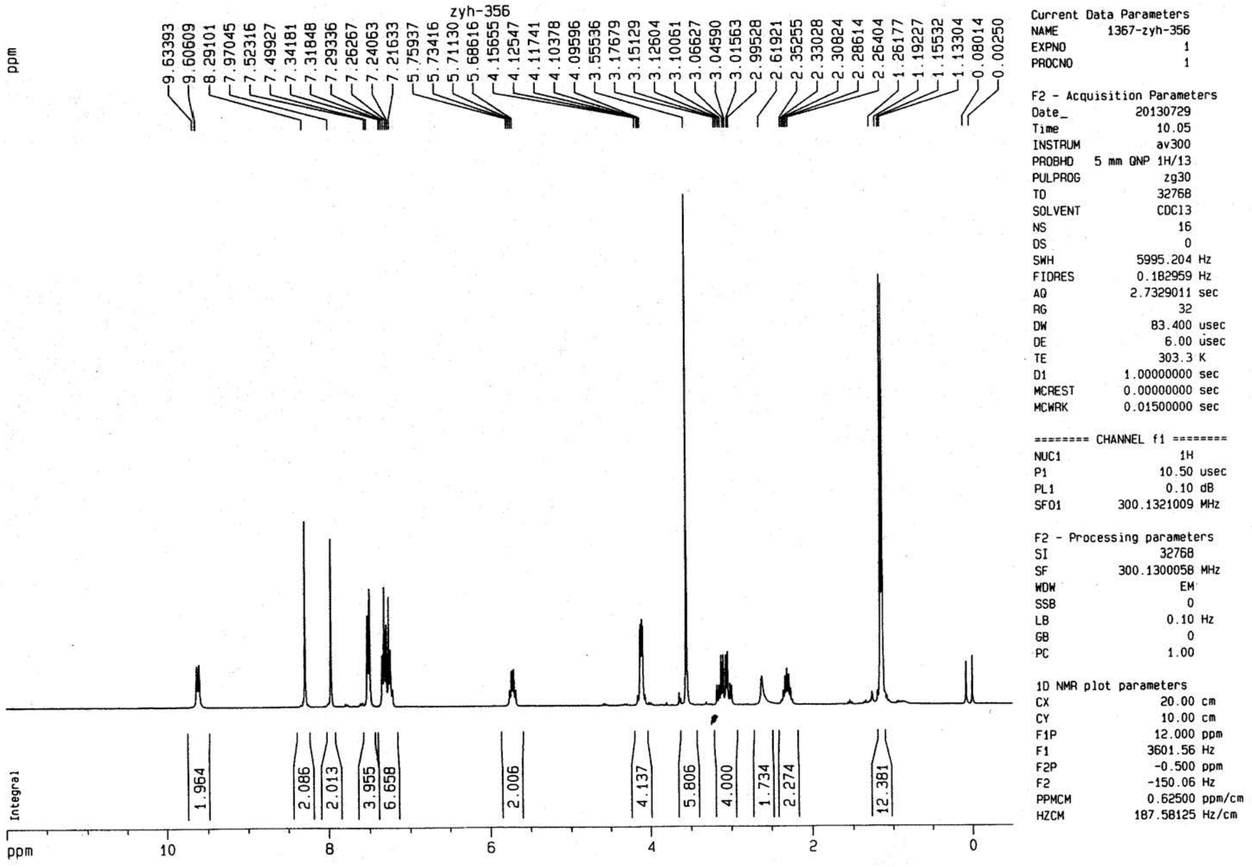

Figure S6. ^{1}H NMR of ***S*-G1** in $CDCl_3$.

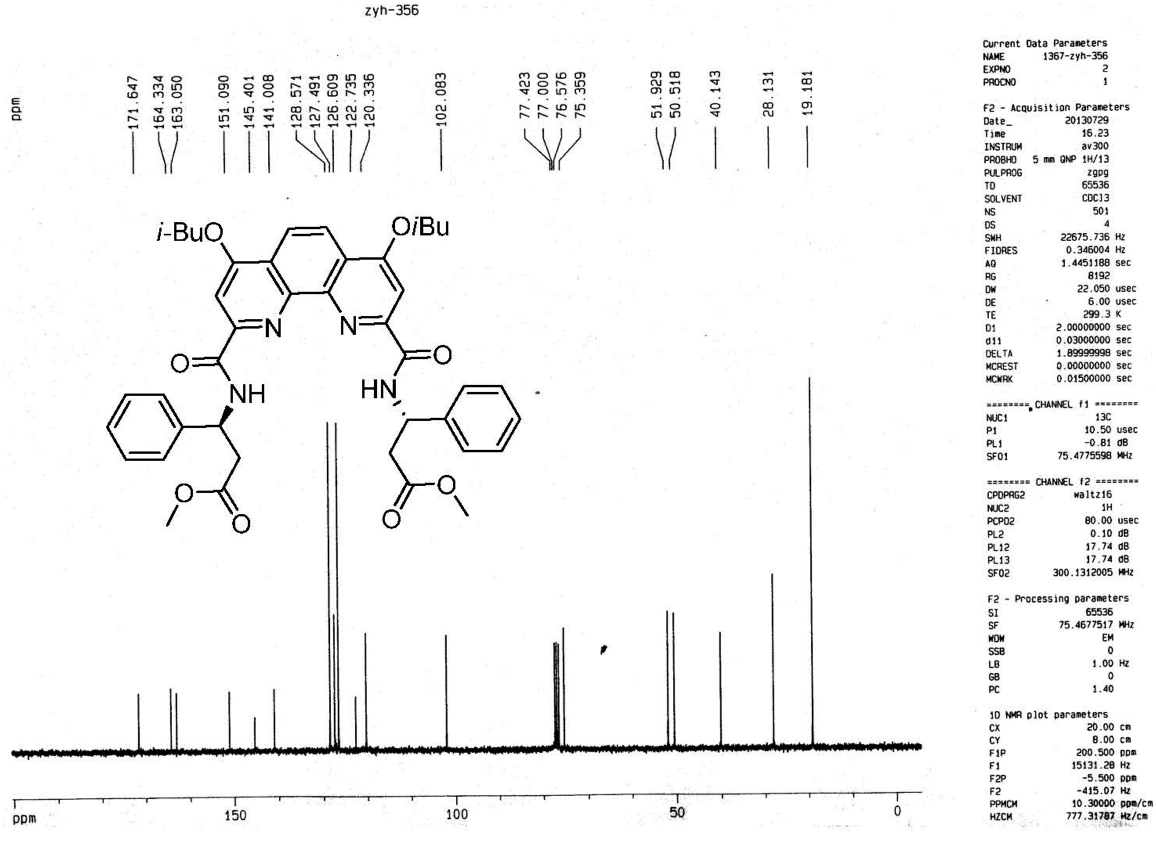

Figure S7. ^{13}C NMR of ***S*-G1** in $CDCl_3$.

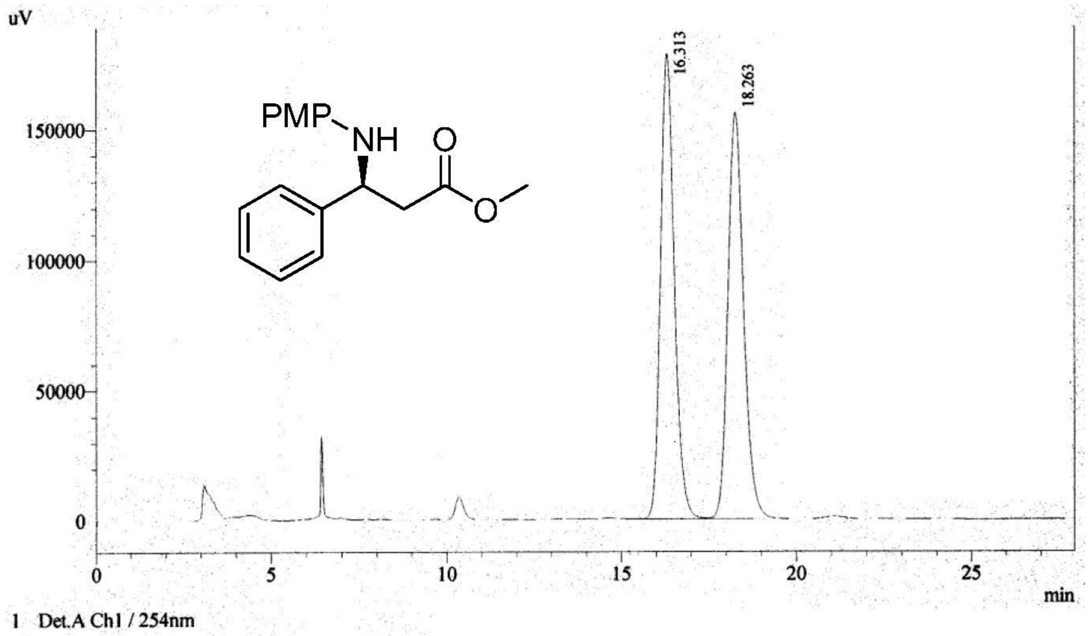

Detector A Ch1 254nm

Peak#	Ret. Time	Area	Height	Area %	Height %
1	16.313	4907847	178797	50.114	53.397
2	18.263	4885446	156046	49.886	46.603
Total		9793293	334843	100.000	100.000

Figure S8. HPLC of racemic **1c**.

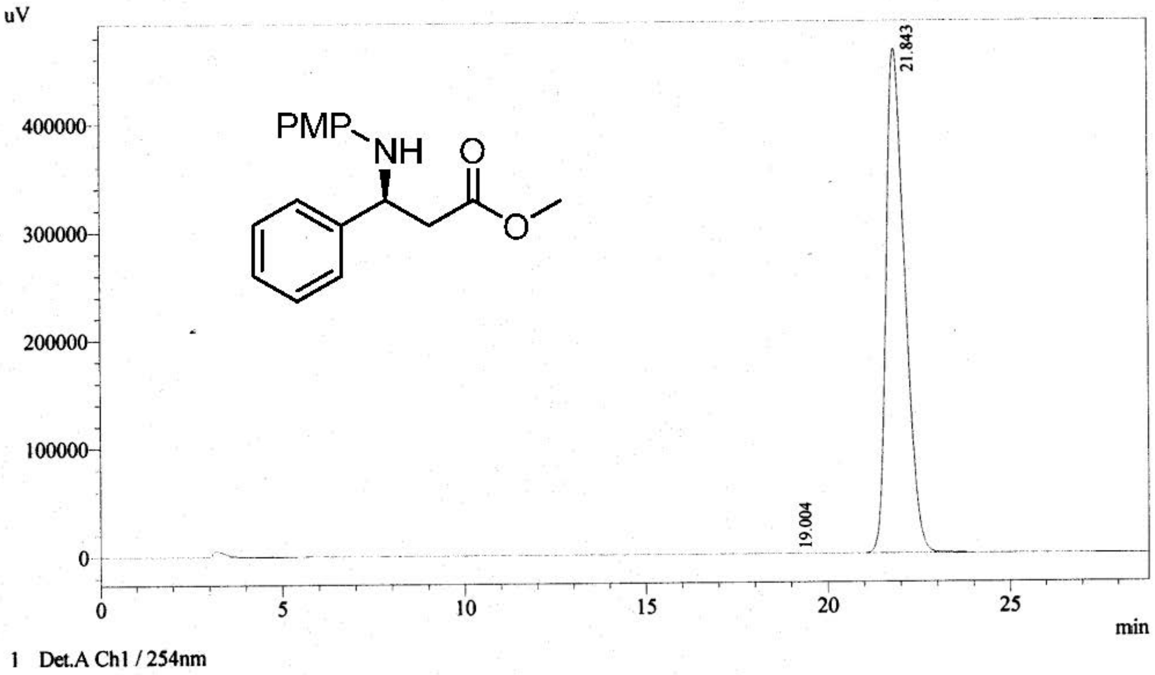

Detector A Ch1 254nm

Peak#	Ret. Time	Area	Height	Area %	Height %
1	19.004	3793	149	0.023	0.032
2	21.843	16654636	466195	99.977	99.968
Total		16658429	466344	100.000	100.000

Figure S9. HPLC of chiral **1c**.

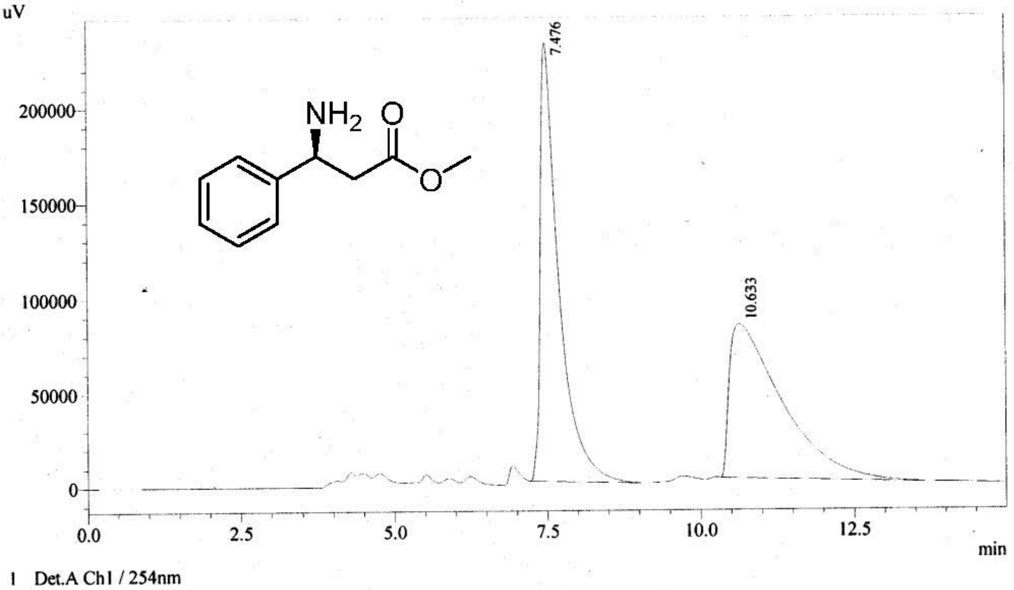

Detector A Ch1 254nm

Peak#	Ret. Time	Area	Height	Area %	Height %
1	7.476	4873998	231628	50.062	74.017
2	10.633	4861972	81312	49.938	25.983
Total		9735969	312939	100.000	100.000

Figure S10. HPLC of racemic ***S*-10**.

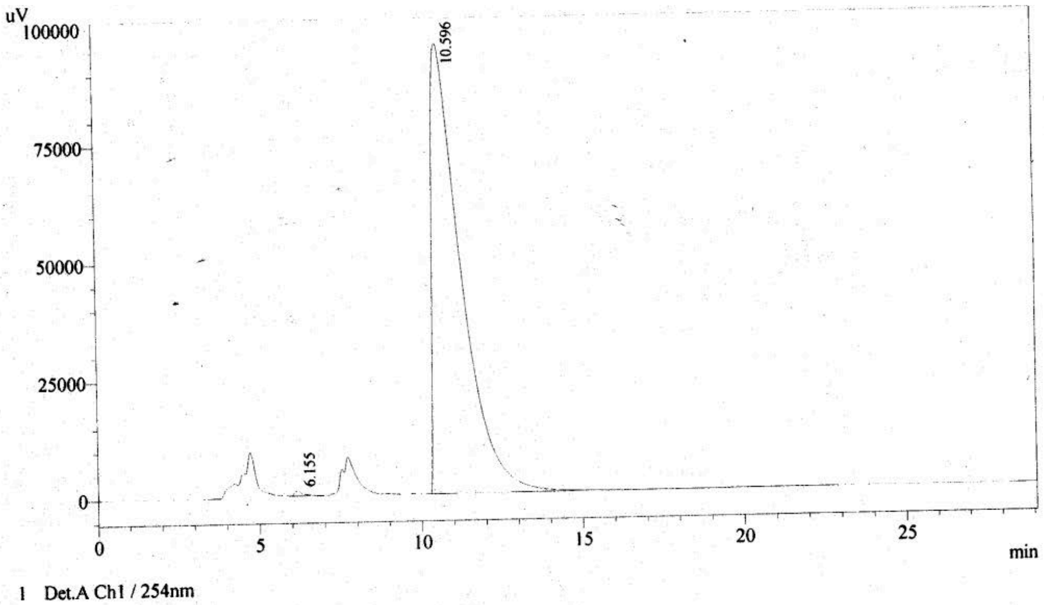

Detector A Ch1 254nm

Peak#	Ret. Time	Area	Height	Area %	Height %
1	6.155	19273	1097	0.325	1.134
2	10.596	5911688	95651	99.675	98.866
Total		5930962	96748	100.000	100.000

Figure S11. HPLC of chiral ***S*-10**.

© 2015 by the authors; licensee MDPI, Basel, Switzerland. This article is an open access article distributed under the terms and conditions of the Creative Commons Attribution license (http://creativecommons.org/licenses/by/4.0/).
